# Genotoxicity of *Euphorbia hirta*: An *Allium cepa* Assay

**DOI:** 10.3390/molecules17077782

**Published:** 2012-06-26

**Authors:** Kwan Yuet Ping, Ibrahim Darah, Umi Kalsom Yusuf, Chen Yeng, Sreenivasan Sasidharan

**Affiliations:** 1 Institute for Research in Molecular Medicine (INFORMM), Universiti Sains Malaysia, 11800 Pulau Pinang, Malaysia; Email: joyc_ping@yahoo.com; 2 School of Biological Sciences, Universiti Sains Malaysia, 11800 Minden, Penang, Malaysia; Email: darah@usm.my; 3 Department of Biology, Faculty of Science, Universiti Putra Malaysia, 43400 UPM Serdang, Selangor, Malaysia; Email: umikay@fsas.upm.edu.my; 4 Dental Research & Training Unit, and Oral Cancer Research and Coordinating Centre (OCRCC), Faculty of Dentistry, University of Malaya, 50603 Kuala Lumpur, Malaysia; Email: chenyeng@um.edu.my

**Keywords:** genotoxicity, *Allium cepa*, ethylmethane sulfonate, mitotic index, chromosome aberrations

## Abstract

The potential genotoxic effects of methanolic extracts of *Euphorbia hirta* which is commonly used in traditional medicine to treat a variety of diseased conditions including asthma, coughs, diarrhea and dysentery was investigated using *Allium cepa* assay. The extracts of 125, 250, 500 and 1,000 µg/mL were tested on root meristems of *A. cepa*. Ethylmethanesulfonate was used as positive control and distilled water was used as negative control. The result showed that mitotic index decreased as the concentrations of *E. hirta* extract increased. A dose-dependent increase of chromosome aberrations was also observed. Abnormalities scored were stickiness, c-mitosis, bridges and vagrant chromosomes. Micronucleated cells were also observed at interphase. Result of this study confirmed that the methanol extracts of *E. hirta* exerted significant genotoxic and mitodepressive effects at 1,000 µg/mL.

## 1. Introduction

Natural products have played a promising role in the treatment and prevention of various diseases throughout the World. Many of the modern drugs available in clinical use todays are of natural product origin. Despite the profound therapeutic advantages possessed by some of the medicinal plants, some constituents of medicinal plants have been found to be potentially toxic, mutagenic, carcinogenic and teratogenic. However, the potential toxicity of herbs has not been recognized by the general public or by professional groups of traditional medicine [1]. This raises concern about the potential toxic effects resulting from the short-term and long-term use of such plants. Therefore, evaluating the toxicological effects of any herbal extract intended to be used in humans is of utmost importance.

*Euphorbia hirta L.* belongs to the family Euphorbiaceae, which is commonly known as asthma weed. The herb is widely used in traditional medicine to treat a variety of diseased conditions including asthma, coughs, diarrhea and dysentery [2]. The sedative, anxiolytic, analgesic, antipyretic and anti-inflammatory properties of *E. hirta* have been reported in the literature [3]. Furthermore, studies revealed that *E. hirta* possess galactogenic, anti-anaphylactic, antimicrobial, antioxidant, anticancer, antifeedant, anti-platelet aggregation, aflatoxin inhibition, antifertility, anthelmintic, antiplasmodial, antiamoebic, antimalarial, and larvicidal activities [4].

Recent studies have shown that long-term exposures to traditional medicinal herbs might be associated with increases in the rates of morbidity and mortality. In addition to systemic toxicity, the possible genotoxicity of herbal plants has been investigated in recent years. The aim of this study was to evaluate the genotoxicity of *E. hirta* extract by using *in vitro* mutagenicity bioassay on mitotic cells in *Allium cepa* root tips.

## 2. Results

Table 1 shows the cytological effects of *E. hirta* extract on root tip cells of *A. cepa*. Exposure of *E. hirta* extract inhibited the mitotic index in a concentration-dependent manner when compared to the mitotic index of 0.509 in the control group. The lowest Mitotic Index (MI) value of 0.299 was recorded for 1,000 µg/mL treated with *E. hirta* extract. 

The mitotic index for *E.* *hirta* extract decreased significantly (*p* < 0.05) at 500 µg/mL and 1,000 µg/mL. The mitotic indexes were 0.379 and 0.299 respectively as compared to mitotic index at 125 µg/mL and 250 µg/mL which were 0.403 and 0.406 respectively. This may indicate that *E. hirta* methanol crude extract exerted a genotoxic effect at 1000 µg/mL. The mitotic indexes in treated cells were lower compared to the distilled water (negative control) which was 0.509. Ethylmethane sulfonate was used as positive control. As shown in Table 1, the mitotic index decreased at the same rate as the concentration increased from 125 µg/mL to 1,000 µg/mL. The lowest MI value for the highest concentration of ethylmethane sulfonate at 1,000 µg/mL is 0.184.

**Table 1:** Cytogenetic analysis of *A. cepa* root tips exposed to different concentrations of *E. hirta* extract and ethylmethane sulfonate.

Treatments			Chromosome aberrations				
Concentration (µg/mL)	No. of cells	Mitotic Index	Stickiness	Bridges	C-mitosis	Vagrant	% of Aberrant cells
***E. hirta***							
125	1059	0.403 ± 0.042 *	3	2	3	4	1.13
250	1124	0.406 ± 0.063 *	4	5	2	4	1.33
500	1045	0.379 ± 0.040 *	2	3	2	14	2.01
1000	1070	0.299 ± 0.035 *	12	18	7	2	3.64
**Ethylmethane sulfonate**							
125	1225	0.410 ± 0.035 *	4	2	5	5	1.31
250	868	0.339 ± 0.035 *	8	3	12	3	3.00
500	1065	0.294 ± 0.049 *	20	5	2	6	3.01
1000	1105	0.184 ± 0.021 *	26	14	28	6	6.70
**Distilled water **	1183	0.509 ± 0.034	0	1	0	1	0.17

* *p* < 0.05 *versus* control (Distilled water).

**Figure 1:**
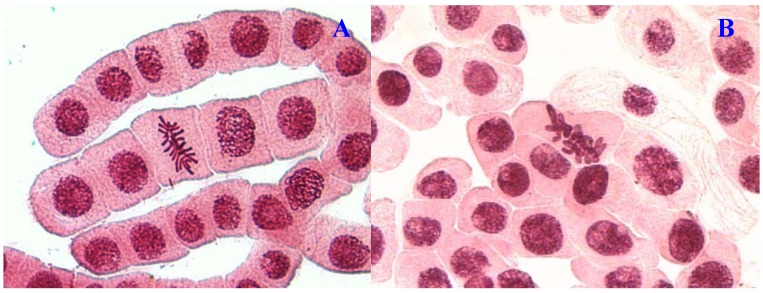
Chromosome aberrations observed in *A. cepa* meristematic cells exposed to methanol extracts of *E. hirta*. (**A**), normal metaphase and (**B**), sticky chromosome. Magnification 400×.

**Figure 2:**
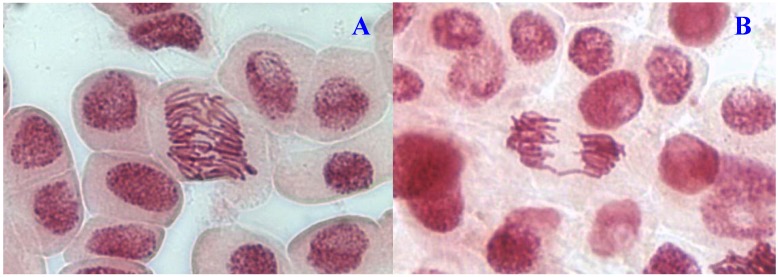
Chromosome aberrations observed in *A. cepa* meristematic cells exposed to methanol extracts of *E. hirta*. (**A**), normal anaphase and (**B**), chromosome bridge. Magnification 400×.

**Figure 3:**
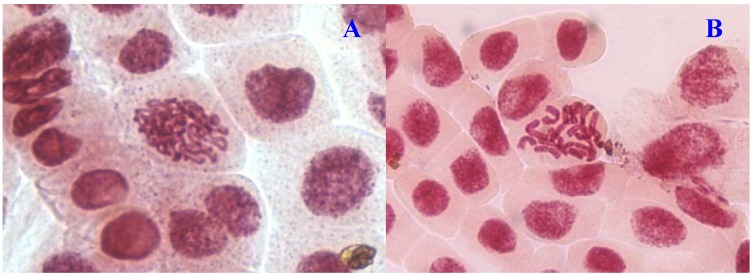
Chromosome aberrations observed in *A. cepa* meristematic cells exposed to methanol extracts of *E. hirta*. (**A**), normal prophase and (**B**), C-mitosis. Magnification 400×.

**Figure 4:**
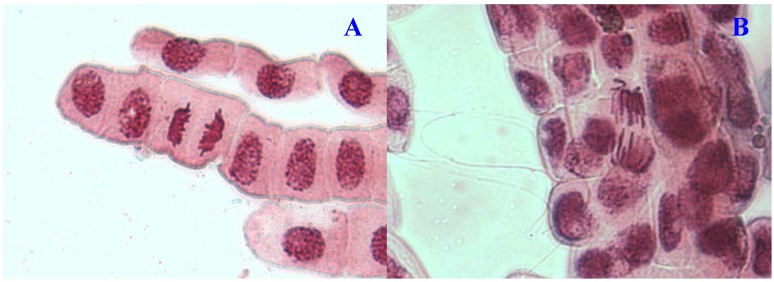
Chromosome aberrations observed in *A. cepa* meristematic cells exposed to methanol extracts of *E. hirta*. (**A**), Normal *telophase* and (**B**), vagrant chromosome. Magnification 400×.

**Figure 5:**
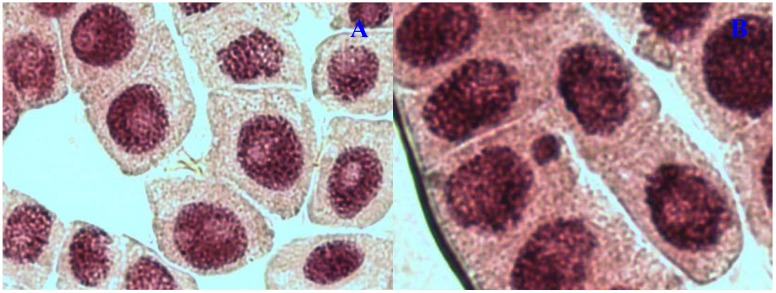
Chromosome *aberrations* observed in *A. cepa* meristematic cells exposed to methanol extracts of *E. hirta*. (**A**), Normal interphase and (**B**), micronucleus in interphase. Magnification 400×.

Chromosome aberrations were observed in all stages of mitosis. Table 1 showed the types and frequencies of chromosome aberrations induced by treatments. *E. hirta* extract showed concentration-dependent increase in the frequency of chromosome aberrations. At high concentration (1,000 µg/mL), sticky chromosomes and chromosome bridges were the most common chromosome aberrations observed (Figure 1B and Figure 2B). Other chromosomal abnormalities observed were c-mitosis and vagrant chromosomes (Figure 3B and Figure 4B). *E. hirta* at 1,000 µg/mL showed half as much % aberrations as compared to positive control. For methylmethanesulfonate, stickiness and c-mitosis were found to be the frequent aberrations observed. In control root tips samples, percentage of aberrations cells is low, 0.17% compared to highest concentration of ethylmethanesulfonate, which scored 6.7%. Micronucleated cells also were observed at interphase (Figure 5B).

The induction of micronucleus formation was generally observed in all tested concentration of *E. hirta* and significantly different when compared with the negative control (*p* < 0.05). Micronucleus formation was markedly higher at 1,000 µg/mL than at the other concentrations (Table 2).

**Table 2:** Genotoxic effects of *E. hirta* extract on cells of *Allium cepa* micronucleus assay.

Treatments		
Concentration (µg/mL)	No. of interphase cells examined	Micronucleus %
***E. hirta***		
125	1520	0.0197% *
250	1543	0.0214% *
500	1818	0.0264% *
1000	1764	0.0272% *
**Ethylmethane sulfonate**		
125	2095	0.0200% *
250	1542	0.0220% *
500	2130	0.0282% *
1000	1743	0.0304% *
**Distilled water **	1674	0.0024%

* *p* < 0.05 *versus* control (distilled water).

## 3. Discussion

Higher plants such as *A. cepa* are accepted as admirable genetic models to evaluate genotoxic effects such as chromosome aberrations and disturbances in the mitotic cycle. Results of the current study reflected the utility of root tips of cells of *A. cepa* for monitoring the genotoxic effects of plant extracts. *A. cepa* assay enabled the assessment of different genetic endpoints, which are mitotic index and chromosome aberration. Mitotic index was characterized by the total number of dividing cells in cell cycle. Mitotic index is used as an indicator of cell proliferation biomarkers which measures the proportion of cells in the mitotic phase of the cell cycle. Hence, the decrease in the mitotic index of *A. cepa* meristematic cells could be interpreted as cellular death. Several types of chromosome aberrations were considered in the four phases of cell division (prophase, metaphase, anaphase and telophase) to evaluate chromosomal abnormalities. According to Rank and Nielsen [5], chromosome aberrations analysis not only allowed estimation of genotoxic effects, but also enabled evaluation of their clastogenic and aneugenic actions. 

Low mitotic index may be reflecting a direct genotoxic effect of *E. hirta* extract. Therefore the mitotic index was analysed in this study do determined the genotoxicity of *E. hirta* extract treatment on *A. cepa*. The cells of *A. cepa* root tips after treatment with extracts of *E. hirta* showed decreased in mitotic index with increasing concentration. There were significant differences (*p* < 0.05) between treated groups and control group in mitotic index (Table 1). The mitotic activity of *E. hirta* methanol extract was significantly decreased at the tested concentrations at 1,000 µg/mL (Table 1), with 41.26% decreased in mitotic index as compared to the control. Ethylmethane sulfonate was used as positive control in this study. A dose dependent decrease of mitotic index was observed in the ethylmethane sulfonate. Treatment with concentration of 1,000 µg/mL showed an absence of dividing cells as the mitotic activity dropped 63.85% as compared negative control. Ethylmethane sulfonate is a genotoxic chemical, where positive results have been consistently reported in numerous *in vitro* mutagenicity and genotoxicity assays.

The mitodepressive effect suggests that *E. hirta* extract had some effects on cell division of *A. cepa*. This may be due to abnormal conditions of the cells induced by the treatments. The abnormalities of chromosomes could be due to the blockage of DNA synthesis or inhibition of spindle formation. *E. hirta* extract may not even allow the initiation of their biosynthesis [6]. The reduction of the mitotic index might be explained as being due to the obstruction of the onset of prophase, the arrest of one or more mitotic phases, or the slowing of the rate of cell progression through mitosis [7]. 

Chromosome aberrations provided important information and may be considered an efficient test to investigate the genotoxic potential of the treatments analyzed [8]. The chromosome aberrations observed at all concentrations of the treatment were chromosome stickiness, bridges, c-mitosis and vagrant chromosomes. These aberrations were due to the effect of the extract on the spindle formation and thus resulted in cell division disturbances. Chromosome bridges indicating the clastogenic effect caused by chromosome breaks, whereas vagrant chromosomes and c*-*metaphases increase the risk for aneuploidy [9]. 

Some of the physiological aberrations that were commonly observed in this study were stickiness (Figure 2B). A remarkable correlation between the frequencies of stickiness and the bridges was observed. This supports the hypothesis that stickiness may result from improper folding of chromosome fibers which makes the chromatids connected by means of subchromatid bridges [10,11]. However, Mercykutty and Stephen [12] reported that this stickiness may be interpreted as a result of depolymerisation of DNA, partial dissolution of nucleoproteins, breakage and exchanges of the basic folded fibre units of chromatids and the stripping of the protein covering of DNA in chromosomes. According to Fiskesjo [13], sticky chromosomes indicated a highly toxic, irreversible effect, probably leading to cell death. 

Another remarkable abnormality was chromosome bridges. Chromosome bridges were commonly observed during anaphase and telophase (Figure 3B). The bridges noticed in the cells were probably formed by breakage and fusion of chromatids or subchromatids [14]. According to Kabarity *et al.* [15], chromosome bridges may be caused by stickiness of chromosomes which made their separation and free movements complete and thus they remained connected by bridges. A low frequency of c-mitosis (Figure 1B) and vagrant chromosomes was also observed. Their presence may be attributed to the failure of the spindle apparatus to organize and function in a normal way. Similar observations have been made by other workers where c-mitosis was regarded as indicative of a weak toxic effect which may be reversible [13]. However, these changes may induce the formation of polyploid cells when not reversed [16]. Vagrant chromosomes that were not organized to a specific stage of the mitotic division were also observed (Figure 4B). This abnormality may be caused by unequal distribution of chromosomes with paired chromatids in which resulted from nondisjunction of chromatids in anaphase. Vagrant chromosomes were weak c-mitotic effect indicating risk of aneuploidy [13].

The chromosome aberration and micronucleus assays have been shown to be highly reliable in genotoxicity testing [17]. Besides to the types of chromosome aberrations, the formation of micronucleus in interphase cells was determined. The percentage of micronucleated cells was obviously higher than control group (*p* < 0.05) at all tested concentration. The induction of micronucleus in root meristem cells of *A. cepa* is the manifestation of fragments or vagrant chromosomes [18]. 

## 4. Experimental

### 4.1. Plant Sample

A sample of *E. hirta* was collected in January 2011, from the Universiti Sains Malaysia campus, Pulau Pinang, Malaysia and authenticated at the Herbarium of the School of Biological Sciences, Universiti Sains Malaysia, Pulau Pinang, Malaysia where a sample (voucher number 11215) has been deposited.

### 4.2. Preparation of the Plant Extract

The plant material was washed with tap water and then with distilled water. The plant material was then dried in an oven at 50 °C for 3 days, after which the dried plant material was ground into fine powder using a grinder. A hundred grams of all plant part powder was extracted by maceration in 400 mL of methanol for 4 days with frequent agitation. The mixture was filtered through clean muslin cloth followed by double filtration with Whatman No. 1 filter paper and the filtrate was concentrated by a rotary evaporator under vacuum at 50 °C, poured in glass Petri dishes and brought to dryness at 60 °C oven. The percentage yield of the crude extract was 17.1%. The obtained paste like mass was then stored in Parafilm-sealed Petri-dishes in dark cabinet. The extract was reconstituted by dissolving in methanol to the required concentrations. 

### 4.3. Allium cepa *Assay*

#### 4.3.1. Pre-Treatment

The *A. cepa* bulbs were grown in tap water at room temperature for 2–3 days. When the roots were 2–4 cm in length, the bulbs were treated with different concentrations of the crude extracts (125, 250, 500, 1,000 µg/mL). Another set of plants was placed in ethylmethane sulfonate (125, 250, 500, 1,000 µg/mL) as positive controls while for the negative control, a set of *A. cepa* was growing in water. The solutions were changed daily and after 48 h, root tips from each bulb was harvested, fixed in Carnoy’s fixative (1:3 acetic acid: alcohol) for 24 h. It was then proceed to slides preparation or stored in 70% alcohol [19].

#### 4.3.2. Slides Preparation

Preparation of slides was carried out as according to Sharma and Sharma [20]. After pre-treatment, the root tips were washed a few times with distilled water. They were hydrolyzed with 1 N HCl at 60–70 °C for 5 min. After hydrolysis, the roots were washed. Then, about 1–2 mm of the root tips were cut and placed on the slide. A small drop of aceto-orcein was dropped on the root tip and left for 2 min. The root tip was then squashed with metal rod and another small drop of aceto-orcein was added and left for another 2 min. The cover slip was carefully lowered on to avoid air bubbles and the sides of the slides were sealed with clear fingernail polish. 

#### 4.3.3. Observation of Specimens

The slides were observed under the light microscope at 400× and 630× magnification. An Olypmus light microscope with digital camera was used in order to get the clear image of the chromosome aberrations. Photomicrographs were made and minimum of 100 cells per slide were analysed (nine slides were observed for each treatment). The mitotic index, micronucleus in interphase and chromosome aberrations in mitotic phases was determined by the examination and counting minimum of 100 cells per slide (nine slides were observed for each treatment). The experiment was replicated three times with three roots for each replicate, therefore, nine slides were prepared for each treatment group. The mitotic index was obtained as follows:

Mitotic index = Number of cells in mitosis/Total number of cells [19].

#### 4.3.4. Statistical Data Analysis

Data obtained from the mitotic index calculation were analysed using Analysis of Variance Technique (ANOVA) at significant level of *p* < 0.05 using SPSS Program Version 17. Duncan’s multiple range test was performed to determine the significant differences between treatments (*p* < 0.05). 

## 5. Conclusion

From the present study it appears that *E. hirta* methanolic extract, which is used frequently in the traditional medicine, clearly exhibits chromotoxic and mitodepressive effects at 1,000 µg/mL, therefore, it is necessary to take precautions when using this extract as an alternative remedy in traditional healing systems. In addition, further cytogenetic studies dealing with clastogenicity and genotoxicity of this extract with more comprehensive genotoxicity assessment in animal model may reveal further interesting results for its usage for human welfare.
